# RSV vaccination in older adults: Addressing vaccine hesitancy using the 3C model

**DOI:** 10.1177/17151635231210879

**Published:** 2023-11-24

**Authors:** Sherilyn K. D. Houle, Melissa K. Andrew

**Affiliations:** School of Pharmacy, University of Waterloo, Waterloo, Ontario; Division of Geriatric Medicine, Dalhousie University, Halifax, Nova Scotia

## Abstract

The first vaccine against respiratory syncytial virus (RSV) targeting older adults was approved for use in Canada in August 2023. As a frequent first point of contact for Canadians seeking advice on vaccination and the most common setting for the administration of influenza vaccines, community pharmacies will also play a role in RSV vaccination efforts. To address vaccine hesitancy confidently and effectively, pharmacists must be equipped with knowledge of the factors that affect a person’s decision on whether to be vaccinated or not. The 3C Model of Vaccine Hesitancy summarizes these as complacency, confidence and convenience. This article introduces the model and describes the often-underrecognized relevance of RSV to older adults, including risk factors and burden of disease. It also reviews the history and status of vaccine development and approval and presents clinical trial data to equip pharmacists to discuss RSV vaccination with older adults who express vaccine hesitancy.

## An introduction to RSV

A surge in respiratory syncytial virus (RSV) cases in Canada in the 2022-2023 respiratory virus season^1^ has generated interest in the virus and preventive measures. Until recently, other than respiratory etiquette, handwashing and avoidance of close contact with those who are ill, the only preventive therapies available were passive immunization with the monoclonal antibodies palivizumab and nirsevimab.^2,3^ Indicated for children younger than 2 years, the National Advisory Committee on Immunization (NACI) recommends their use only for those at high risk of complications from RSV due to prematurity (birth at less than 30 weeks’ gestation) or medical conditions such as chronic lung disease, cystic fibrosis, hemodynamically significant congenital or other chronic heart disease or immunosuppression.^4^

While much attention has been given to RSV among the pediatric population, its morbidity and mortality in older adults is also significant. One limitation to current knowledge is underdiagnosis, as RSV testing has not been routinely implemented in community settings, and inconsistently among hospitalized cases of adult acute respiratory illness (ARI). In addition, antigen tests that may be used in the primary care setting are better able to detect illness in young children (who have a greater viral load in the upper respiratory tract) versus older adults who, at least in cases of more severe and clinically relevant illness, tend to express the virus in the lower respiratory tract.^5^ Even so, the burden of RSV is estimated to be significant, causing 8400 deaths annually among adults aged ≥65 years in the United Kingdom,^5^ while a systematic review on RSV in adults aged ≥60 years estimated that there are >5.2 million cases, 470,000 hospitalizations and 33,000 in-hospital deaths annually in high-income countries.^6^ RSV can also lead to complications and persistent health problems. For example, RSV illness in older adults has been associated with pneumonia, exacerbation of chronic disease, worsening of frailty and an elevated risk of death persisting more than 1 year following infection.^7,8^ Analogously, influenza is known to increase the risk of cardiovascular events^9,10^ as well as exacerbations of underlying comorbidities such as chronic obstructive pulmonary disease (COPD)^11^ and diabetes.^12^ While comparable outcomes are suspected to occur in relation to RSV infection, this remains an important knowledge gap. These risks of poor outcomes following RSV infection become heightened above age 60 years and are compounded by the presence of other risk factors such as COPD, functional impairment and residence in long-term care facilities.^5,13^ Notably, illness presentation overlaps with other infectious and noninfectious causes of ARI, so a high index of suspicion is clinically warranted to avoid underdetection.

**Knowledge into Practice**

- Awareness of RSV has risen following a surge in cases and resulting hospitalizations in 2022-2023; however, the public and many health professionals still view RSV as primarily a childhood disease.
- RSV vaccination in older adults aims to reduce morbidity and mortality in this population, which is at an elevated risk of complications from RSV.
- The 3C Model of Vaccine Hesitancy has been applied to efforts to identify and address concerns about many vaccines. This article provides pharmacists with information and strategies to address vaccine hesitancy specific to RSV in practice.

To reduce this burden of disease, research and development of vaccines and therapeutics for RSV has seen rapid growth, with numerous products at various stages of clinical trials and approval, including nucleic acid, subunit and vector-based vaccines.^14^ As of November 2023, one RSV vaccine (recombinant, adjuvanted) has been approved by Health Canada for use in adults aged ≥60 years under the trade name Arexvy,^15^ with another under review seeking indications for use in adults aged ≥60 years and for maternal immunization to provide protection to the newborn.^16^

It is well known that the availability of an approved vaccine does not guarantee its uptake by the target population. Apart from the high uptake seen with COVID-19 vaccinations among older adults (>95% among Canadian adults aged 70-79 years and >99% among those aged ≥80, for completion of the primary series),^17^ uptake among older adults (aged ≥65 years unless otherwise specified) for other routinely recommended immunizations is consistently below Public Health Agency of Canada vaccination rate targets of ≥80% for influenza (70.4%), pneumococcal disease (54.8%), tetanus (51.8%), pertussis (23.7%) and shingles (27.4%, among adults aged ≥50 years).^18,19^ While the causes are likely multifactorial, vaccine hesitancy is a contributing factor, with Canadian pharmacists reporting encountering an average of 16 individuals expressing vaccine hesitancy weekly during the influenza season,^20^ prior to the COVID-19 pandemic. As vaccine hesitancy spans all demographics, products and vaccine-preventable diseases, it is to be expected that pharmacists will also encounter some hesitancy around RSV vaccination. This article aims to equip pharmacists with the knowledge and tools to address RSV-related vaccine hesitancy in practice.

**Mise En Pratique Des Connaissances**

- La sensibilisation au virus respiratoire syncytial (VRS) s’est accrue à la suite d’une augmentation soudaine des cas et des hospitalisations qui ont eu lieu en 2022/2023. Cependant, le public et de nombreux professionnels de la santé considèrent encore le VRS comme une maladie principalement infantile.
- La vaccination contre le VRS chez les personnes âgées vise à réduire la morbidité et la mortalité au sein de cette population, qui présente un risque élevé de complications dues au VRS.
- Le modèle des 3C de l’hésitation vaccinale a été appliqué aux efforts visant à identifier les préoccupations sur de nombreux vaccins, et à y répondre. Cet article fournit aux pharmaciens des informations et des stratégies pour remédier à l’hésitation vaccinale spécifique au VRS dans la pratique.

## The 3C Model of Vaccine Hesitancy

Vaccine hesitancy has been defined by the World Health Organization as the “delay in acceptance or refusal of vaccines despite availability of vaccination services.”^21^ The 3C Model of Vaccine Hesitancy considers complacency, confidence and convenience to be contributing factors, as defined below^22^:

• **Complacency:** Occurs when perceived risks of vaccine-preventable diseases are low and vaccination is not deemed a necessary preventive action.
• **Confidence:** Consists of trust in (1) the effectiveness and safety of vaccines; (2) the system that delivers them, including the reliability and competence of the health services and health professionals; and (3) the motivations of the policy makers who decide on the needed vaccines.
• **Convenience:** The extent to which physical availability, affordability and willingness to pay, geographical accessibility, ability to understand (language and health literacy) and appeal of immunization services affect uptake.^22^

This model has been found to be applicable to various populations and vaccine products.^23-27^ Below, we apply it to the unique considerations for RSV vaccination in the older adult population.

## Complacency and RSV vaccination

Media reports on RSV in the 2022-2023 respiratory season focused on its incidence among children and the resulting burden on primary care and hospital services.^28-30^ Given this and the historical focus on young children in the research and clinical literature reviewed above, it is unsurprising that both clinicians and the public view RSV as largely a childhood concern. While common among children (serologic studies in Finland have estimated that 86% of the population has been infected by the age of 3 years)^31^ and the leading cause of hospitalization in infants,^32^ hospitalization and mortality counts are notably higher among adults ≥65 years (177,000 versus 58,000 hospitalizations and 14,000 versus 100-500 deaths annually in the United States).^32^

Hospitalization rates are also significantly higher among older adults with comorbidities such as asthma, coronary artery disease, diabetes, COPD and congestive heart failure, with incidence rate ratios ranging from 2.27 for asthma to 13.41 for COPD versus those without the condition.^33^ A systematic review of studies in the United States found that older adults hospitalized with RSV typically experience a length of stay of 3 to 6 days, with 10% to 31% requiring intensive care and 3% to 17% requiring mechanical ventilation and a mortality rate of 6% to 8%.^34^ Other research has similarly concluded that hospitalization for RSV results in similar lengths of stay, rates of use of intensive care and mortality as influenza A.^7^ Beyond hospitalization, complications from RSV can persist, with 23% of older adults requiring a higher level of care after discharge.^35^ This is in comparison to research from California that has found a lower 1-year survival rate after admission for RSV than for influenza (74.2% versus 81.2%, *p* < 0.001).^36^

Each year, 7 in 10 Canadian older adults choose to be vaccinated against influenza,^37^ an indication that the majority view the consequences of influenza to be a greater risk than the potential for any adverse events associated with vaccination. Educating the public that RSV is associated with similar, if not worse, rates of complications as influenza in older adults may help overcome complacency about the value of RSV vaccination in this population. Expressing the benefits of vaccination in terms that matter to an older person (e.g., a way to maintain functional independence and framing it as a way to *avoid* frailty, as has been the message of the Canadian Frailty Network)^38^ can be helpful to convey the benefits beyond preventing an acute illness, which might be seen as a lesser priority.

## Confidence and RSV vaccination

As with all vaccines, candidate RSV vaccines must successfully progress through 3 stages of clinical trials involving thousands of volunteers which examine optimal dosing, adverse effects and efficacy before receiving approval for use.^39^ This clinical trial data are made publicly available for submissions to the US Food and Drug Administration (FDA) but are not made available for drugs awaiting approval with Health Canada. As such, at the time of writing, according to the Canadian product monograph for Arexvy^40^ and the clinical trial data for the vaccine under Health Canada review published in its US monograph,^41^ clinical trials reported adverse effects similar in frequency and severity as other available vaccines similar in composition (e.g., local reactions such as pain, redness and swelling and systemic reactions such as fatigue, headache and myalgia).

Patients may have questions about the risk of Guillain-Barré syndrome (GBS) following RSV vaccination, as clinical trials reported 1 to 2 cases among 15,000 to 20,000 participants,^40,41^ which is higher than the background rate for GBS among adults ≥60 years of age of 1.85 to 2.66 cases per 100,000 people per year.^42^ This finding contributed to split decisions by FDA Advisory Committees on the approval of these vaccines^43,44^ and accompanying recommendations to monitor for GBS specifically in postmarketing surveillance studies. GBS has been reported in association with other viral infections such as influenza^45^ and COVID-19,^46^ with a single case report published to date on GBS following infection with RSV.^47^ Additional research is needed to compare GBS rates among those receiving RSV vaccination versus natural infection in larger populations.

Some may also express discomfort with RSV vaccination related to a failed initial attempt to develop a vaccine in the 1960s, where an inactivated RSV vaccine was administered to infants who were then exposed to naturally circulating RSV in the community. Of 31 infants receiving the vaccine, 20 were infected during an outbreak, of whom 16 were hospitalized and 2 died.^48^ It was discovered that the children who received the RSV vaccine experienced enhanced respiratory disease (ERD) due to the generation of a nonprotective antibody response that also resulted in eosinophil and immune complex deposition in the lungs.^49,50^

Further research has identified that the F glycoprotein on the surface of the virus exists in 2 conformations, known as prefusion F (preF) and postfusion F (postF).^48^ As the names suggest, the preF conformation exists before fusion with a host cell, and, upon fusion, the protein changes in structure into the postF conformation. This is important to understand, since antibodies that exclusively bind to preF have much greater neutralizing potency than those that bind to both preF and postF.^48^ Unfortunately, when it comes to manufacturing RSV vaccines, preF is highly unstable and will spontaneously rearrange into postF, which has much greater stability. This is likely what happened with the vaccine developed in the 1960s, explaining why recipients did not mount an effective immune response. It took more than 40 years for scientists to understand and stabilize the preF protein,^51^ which is the antigen contained in current RSV vaccines. Patients can be reassured that no cases of ERD occurred in clinical trials of the Health Canada–approved vaccine^40^ or the additional vaccine under review,^41^ with serious adverse event rates nearly identical between vaccine and placebo groups. Older children and adults are also at lower risk of ERD overall than infants in their first RSV season due to priming by prior natural RSV infection.^49^

While safety is an important consideration affecting confidence, effectiveness is also factored into patients’ risk/benefit evaluation. The RSV vaccine currently approved in Canada has reported 82.6% (95% confidence interval [CI] 57.9-94.1) efficacy against any RSV lower respiratory tract disease and 94.1% (95% CI 62.4-99.9) against severe disease in the first RSV season.^40^

## Convenience and RSV vaccination

To date, the approved and submitted RSV vaccines in Canada are for single-dose regimens.^40,41^ While a clinical trial is ongoing (ClinicalTrials.gov identifier NCT04732871) to evaluate the persistence of protection from Arexvy, interim results have found no benefit to a booster dose given 1 year after an initial dose versus the initial dose alone for protection across 2 RSV seasons.^52^ This suggests that vaccination against RSV will likely not be an annual recommendation as it is with influenza. Clinical trials have also established the safety and effectiveness of coadministration with influenza vaccines including standard-dose, high-dose and adjuvanted products.^40^ While NACI has yet to publish advice on vaccine coadministration, as stated by the Advisory Committee on Immunization Practices in the United States, coadministration of RSV vaccine with other vaccines, including those against COVID-19, pneumococcal disease, tetanus and diphtheria with or without pertussis, and herpes zoster is acceptable.^53^ Both the duration of protection of the vaccine across at least 2 seasons and the option of coadministration can be expected to positively affect the convenience of being vaccinated against RSV.

It is also recognized that the ability to be vaccinated at a pharmacy offers the public convenience, flexibility and the ability to be vaccinated close to home; yet, it is a service that is inconsistently available across the country.^54,55^ To achieve the greatest benefit for patients and society, it is imperative that RSV vaccinations be made available through community pharmacies, including the ability for pharmacy professionals to administer the vaccine, to access a publicly funded supply (if added to provincial/territorial routine immunization programs) and to prescribe the vaccine if required. Administration by trained pharmacy students and regulated pharmacy technicians will further enhance the ability of community pharmacies to offer RSV vaccination services. Pharmacy regulatory and advocacy organizations are encouraged to address scope, access and remuneration barriers that may impede RSV vaccination in pharmacies.

## Putting it into practice

This article reviews the clinical importance of RSV in older adults (both as a cause of ARI that can require hospitalization and has significant mortality and as a cause of persistent functional decline and other important long-term health complications) and the benefits of vaccination. Table 1 summarizes key points pharmacists can incorporate into discussions with patients expressing hesitancy toward vaccination against RSV.

**Table 1:** Key points for addressing RSV vaccine hesitancy using the 3C Model

Model component	Key points
Complacency	• RSV contributes to 177,000 hospitalizations and 14,000 deaths among adults ≥65 years annually in the United States, with elevated risk among those with comorbid chronic disease. • Hospitalization for RSV results in similar lengths of stay, rates of use of intensive care and mortality as influenza A, with 23% of older adults requiring a higher level of care after discharge.
Confidence	• Clinical trials of subunit RSV vaccines have reported adverse effects similar in frequency and severity as other available vaccines, with most reactions being mild/moderate and of short duration. • Guillain-Barré syndrome (GBS) will be closely monitored in postmarketing surveillance studies. It is currently unknown how rates of GBS following vaccination compare with natural infection with RSV. • Current RSV vaccines contain a stabilized form of the preF glycoprotein antigen, which mounts a more effective immune response than the vaccine trialed in the 1960s.
Convenience	• The adjuvanted RSV vaccine has demonstrated a duration of protection spanning at least 2 respiratory virus seasons following a single dose. • Coadministration with influenza vaccines has been found to be safe and effective, with the Advisory Committee on Immunization Practices in the United States advising that coadministration with a number of other vaccines is also acceptable. • Uptake by the public can be enhanced if pharmacy scope, access to publicly funded supply and remuneration are optimized to make RSV vaccination conveniently accessible through community pharmacies.
